# Synthesis and Characterization of Macrocyclic Polyether *N,N*′-Diallyl-7,16-diaza-1,4,10,13-tetraoxa-dibenzo-18-crown-6

**DOI:** 10.3390/molecules21020171

**Published:** 2016-01-29

**Authors:** Julius Toeri, Marie-Pierre Laborie

**Affiliations:** 1 Freiburg Materials Research Center, Stefan-Meier-Straße 21, 79104 Freiburg, Germany; marie-pierre.laborie@biomat.uni-freiburg.de; 2 Chair of Forest Biomaterials, University of Freiburg, Werthmanstr. 6, 79085 Freiburg, Germany

**Keywords:** *N*-substituted azacrown polyether, 4,16-diaza-18-crown-6, synthesis, lariat azacrown ether, molecular recognition

## Abstract

In this study an efficient and direct production procedure for a macrocyclic polyether *N,N*′-diallyl-7,16-diaza-1,4,10,13-tetraoxa-dibenzo-18-crown-6 from the reaction of catechol and *N,N*-bis(2-chloroethyl)prop-2-en-1-amine in *n*-butanol in the presence of a strong base is reported. The synthesis involves a two-step addition of sodium hydroxide to enhance the cyclization process, and at the end of the reaction, the reaction mixture is neutralized and the solvent replaced with water *in-situ* through distillation to afford a relatively pure precipitate that is easily recrystallized from acetone. The yield of the macrocycle was 36%–45% and could be scaled-up to one-mole quantities. The structure and purity of this compound was verified on the basis of elemental analysis, IR, UV-Vis, ^1^H-, ^13^C-NMR, 2D-NMR, mass spectroscopy, and thermal analysis. The white crystalline compound has a sharp melting point of 124 °C and a crystallization temperature of 81.4 °C determined by differential scanning calorimetry. Our motivation behind the synthesis of the bibracchial lariat azacrown polyether ligand was to examine its possible applications in ion-selective polymer-supported materials.

## 1. Introduction

Macrocyclic compounds containing oxygen and nitrogen as donor atoms have been extensively investigated for their ability to form stable complexes with ions within their central cavity [1,2,3,4,5,6]. In the research oriented towards the application of these lariat azacrown ether compounds, a special emphasis has been directed at finding suitable chemical compounds that can selectively bind metal ions [7]. It has been established that nitrogen-containing crown ethers have stronger complexation properties for heavy-metal ions than all-oxygen crowns, which strongly complex alkali and alkaline earth metal ions [1,4,8]. The nitrogen-containing lariat azacrowns were also found to possess enhanced complexing ability for ammonium salts and transition-metal ions over the all-oxygen crown compounds, partly due to differences in polarizability [3,4,8,9,10]. These selective complexing properties have influenced their incorporation into polymeric matrices to produce unique and novel polymer-supported crown ethers [9,11,12,13,14,15]. This is normally achieved by tailoring the macrocycle with appropriate side arms that covalently bind to the prospective polymer backbone. However, practical applications of these compounds are still limited due to the complexity of reactions used in the synthesis and the yields of the isolated products often being moderate [3,5,6]. For instance, the *N,N*′-Bis(substituted) derivatives of 4,13-diaza-18-crown-6 polyether macrocycle are prepared via the macrocycle ring closure reactions with the participation of secondary amine (“pre-formed side-arm” method) [5]. However, this method often leads to contamination by difficult-to-remove inorganics and the formation of linear and lager cyclic byproducts that cause their instability and difficulty in purification [5]. Moreover, in spite of the popularity of these macrocycle compounds based on the proven ability to achieve a strong, selective, and dynamic three-dimensional binding of metal cations, most authors report their synthesis in the scale of a few grams or milligrams normally not sufficient for further application processes [16].

Of particular interest is the synthesis of macrocycle polyether *N,N*′-diallyl-7,16-diaza-1,4,10,13-tetraoxa-dibenzo-18-crown-6. The synthesis of this macrocycle as a potential metal ion chelating ligand in a polymer-supported network has been reported previously [17], albeit the yield and purity were not documented. Our attempts at exactly reproducing this procedure gave 6% yield or less of the targeted compound. Therefore, the method was not found to be particularly versatile.

In the present paper, we report for the first time the synthesis of this macrocyclic polyether directly from catechol and the precursor compound *N,N*′-bis(2-chloroethyl)prop-2-en-1-amine in a scaled-up batch size of up to one mole of reactants (corresponding to a product mass of *ca*. 91 g). The purpose of this work was therefore to improve the overall efficiency and yield of this synthesis and increase the batch size to one mole of the reactants. In addition to the optimized and up-scaled procedure, we provide a full structural and physical characterization of the product including ^1^H-, ^13^C-, and 2D-NMR as well as mass spectroscopy, FT-IR, UV-Vis and thermal analysis to support the proposed molecular structure and its purity. By establishing the spectroscopic properties of the macrocyclic polyether and the reaction pathway that leads to improved yields, it is hoped that this compound can find more widespread use in ion-selective materials.

## 2. Results and Discussion

**Figure 1:**
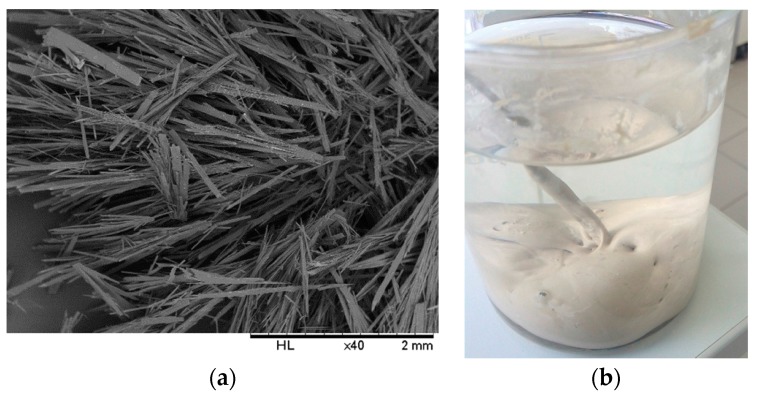
(**a**) SEM image of the azacrown polyether showing fibrous crystals after crystallization (**b**) a photograph of the synthesized azacrown ether precipitate in distilled water before crystallization.

**Scheme 1:**
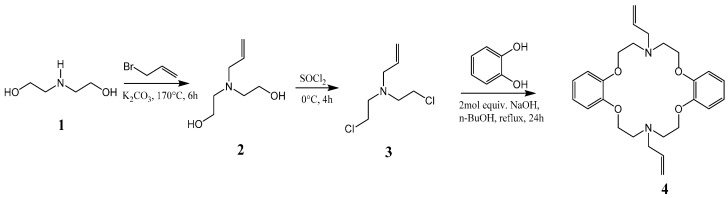
Synthesis of the azacrown polyether.

Bis(2-hydroxyethyl)amine (**1**) is reacted with allyl bromide to form 2,2′-(prop-2-en-1-ylimino)diethanol (**2**), which is then chlorinated to yield the precursor *N*,*N*′-bis(2-chloroethyl)prop-2-en-1-amine (**3**), followed by reaction with catechol in dry *n*-butanol in the presence of a strong base to afford the desired product *N*,*N*′-diallyl-7,16-diaza-1,4,10,13-tetraoxa-dibenzo-18-crown-6 (**4**), as shown in Scheme 1. The macrocycle was reported to have been previously prepared from catechol and the precursor compound **3** [17]. The cyclization reaction involved the generation of catecholate salt by addition of 2-mol. equivalent of sodium hydroxide in *n*-butanol before the slow addition of **3** under reflux to yield the desired product. In our experience, this method affords low yields and the crude product was not easily crystallized, suggesting the presence of impurities. We were able, through some modifications, to improve the overall efficiency of this process and scale up the synthesis to afford the desired product as white fibrous crystals (Figure 1a).

First, it has been proposed that the ring closure reaction step is facilitated by the presence of sodium ion, which is solvated by the intermediate acyclic polyether [18]. In previous studies, it was concluded that the “coordination template effect” is operating and the probability of the ring-closing step is greatly increased by the sodium ion [19]. The results of further experiment appeared to support this assumption. We found yields to be poor in experiments where we added 2 mol. equivalent of sodium hydroxide to catechol/butanol solution all at once. The yields were significantly improved when two aliquots of 1 mol. equivalent of sodium hydroxide was added in two steps, according to Pedersen [18]. Therefore it was assumed that the high yields are ascribed to the sodium ion which is a strong complexer [20] to a “template effect” involving a cation which is not too bulky guiding together the termini of an intermediate, thus increasing the rate of cyclization. Secondly, after refluxing for 21 h, the reaction is neutralized with concentrated hydrochloric acid and the solvent distilled off while it is slowly replaced with distilled water. This step was critical in obtaining the crude product in relatively pure form (Figure 1b) especially when scaling up the production by avoiding an oily phase that is difficult to crystallize. Reactant amounts and yields according to both the previously reported method and the present procedure are included in Table 1. In addition to the improved and scaled up procedure of synthesizing the macrocycle, its spectroscopic and thermal properties have not been reported in detail before, as described in the following sections.

**Table 1:** Comparison of the reaction amounts and the respective yields of the azacrown according to two procedures.

Catechol (Moles)	Moles of 3	Method of Adding NaOH	Yield (%)
11.0 g (0.10 mol.)	0.10	One step 2-mol equivalent	5 ± 1
110.11 g (1.00 mol.)	1.00	One step 2-mol equivalent	3–6
11.0 g (0.10 mol.)	0.10	Two step 1-mol equivalent	36
110.11 g (1.00 mol.)	1.00	Two step 1-mol equivalent	42

### 2.1. Spectroscopic Characterization of the Azarown Polyether

The structures of compounds **2**, **3** and **4** were completely assigned by ^1^H-, ^13^C-NMR and 2D-NMR spectroscopy and supported with infrared signal assignments. The IR spectroscopic data (Figure S1) indicate that the precursor compounds and the synthesized title compound were formed [21,22,23]. 

The UV-Vis spectrum of **4** (Figure S2) was recorded in cyclohexane (2.28 × 10^−4^ M). The product has two UV maxima of 236 nm (ε 12368) and 278 nm (ε 11184) both of which are π→π* and can be attributed to the benzene fragment of the crown ether cavity. A related macrocycle, dibenzo-18-crown-6 polyether, which was synthesized by Pedersen [18], and has all-oxygen crown ether cavity was reported with similar absorption values hence supporting the possibility that the two signals arise as a result of the presence of benzene ring.

**Figure 2:**
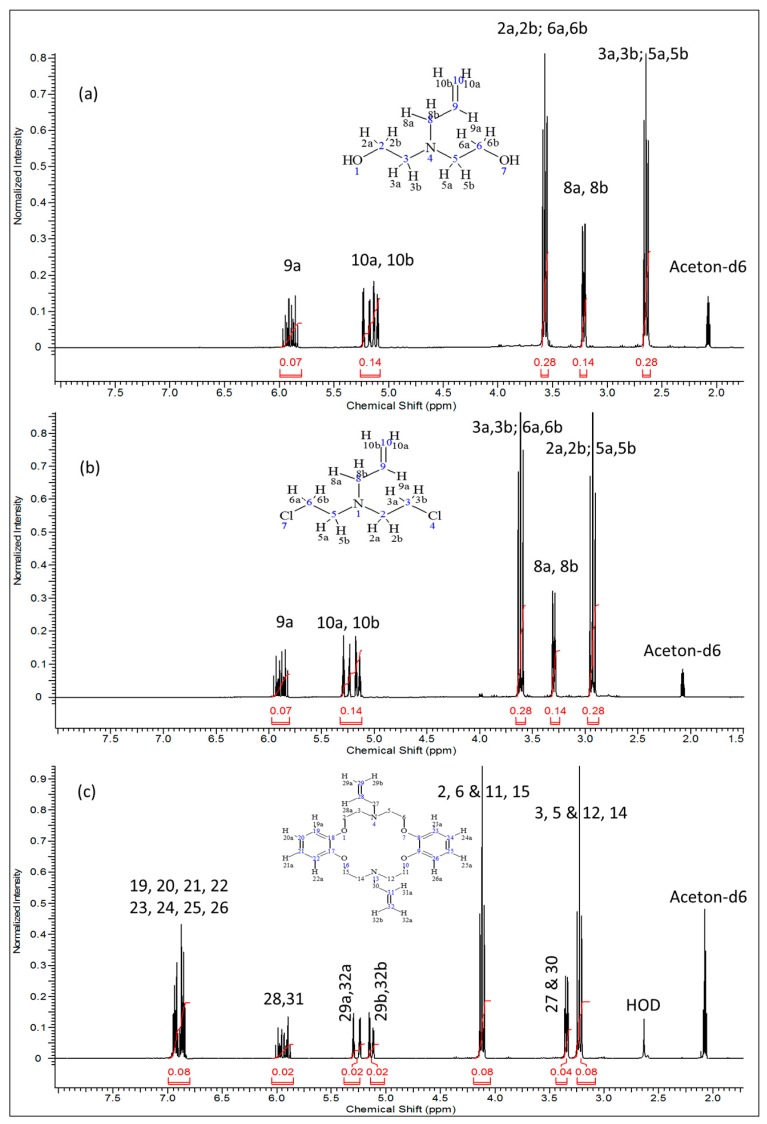
^1^H-NMR spectra of: (**a**) 2,2′-(prop-2-en-1-ylimino)diethanol (**2**) (**b**) *N*,*N*′-bis(2-chloroethyl)prop-2-en-1-amine (**3**); and (**c**) *N,N*′-diallyl-7,16-diaza-1,4,10,13-tetraoxa-dibenzo-18-crown-6 (**4**).

**Figure 3:**
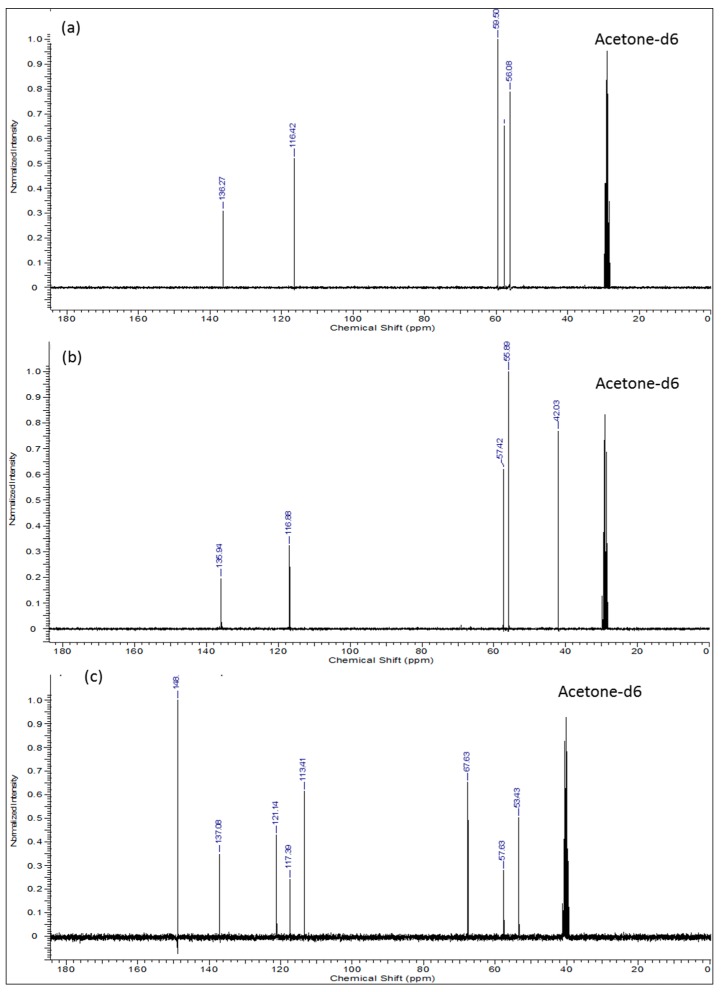
^13^C-NMR spectra of: (**a**) 2,2′-(prop-2-en-1-ylimino)diethanol (**2**); (**b**) *N,N*-bis(2-chloroethyl)prop-2-en-1-amine (**3**) and (**c**) *N,N*′-diallyl-7,16-diaza-1,4,10,13-tetraoxa-dibenzo-18-crown-6 (**4**).

The ^1^H-, ^13^C-NMR and the heteronuclear single quantum coherence (HSQC)-NMR spectra of the synthesized compound were consistent with the assigned structure in all cases. In the proton-NMR spectra (Figure 2), compounds **2** and **3** displayed very well defined resonance signals for the -CH_2_-CH=CH_2_ allyl and -CH_2_CH_2_-X (X = OH or Cl) aliphatic units corresponding to the typical signals of the precursor compounds. In compound **2** (Figure 2a), the *cis* (δ = 5.08–5.15 ppm; m, *J* = 19.9 Hz, 1H_10a_) and the *trans* (δ = 5.20 ppm; dq, *J* = 17.22, 1.71 Hz, 1H_10b_) protons are observed, while the signal at δ = 5.90 ppm (m, *J* = 17.00 Hz, 10.35 Hz, 6.57 Hz, 1H_9a_) represents the single proton of C9. The two equivalent protons at C8 are clearly represented by the signal at 3.21 ppm (dt, *J* = 6.42 Hz, 1.32 Hz, 2H). The four equivalent protons next to the more electronegative nitrogen atom (H_3a,3b_ & H_5a,5b_) are observed at δ = 2.65 ppm (*J* = 5.74 Hz, 4H), while the other four equivalent protons next to –OH groups (H_2a,2b_ & H_6a,6b_) appear downfield at δ = 3.57 ppm (t, *J* = 5.74 Hz, 4H). Integration of the proton signals as shown in Table 2 was consistent with the population. The corresponding ^13^C-NMR spectrum of compound **2** (Figure 3a) indicates the expected five carbon environments and their correct positions, *i.e.*, 136.27 ppm (C9), 116.4 ppm (C10), 59.50 ppm (C3 & C5), 57.8 ppm (C8), 56.1 ppm (C2 & C6).

**Figure 4:**
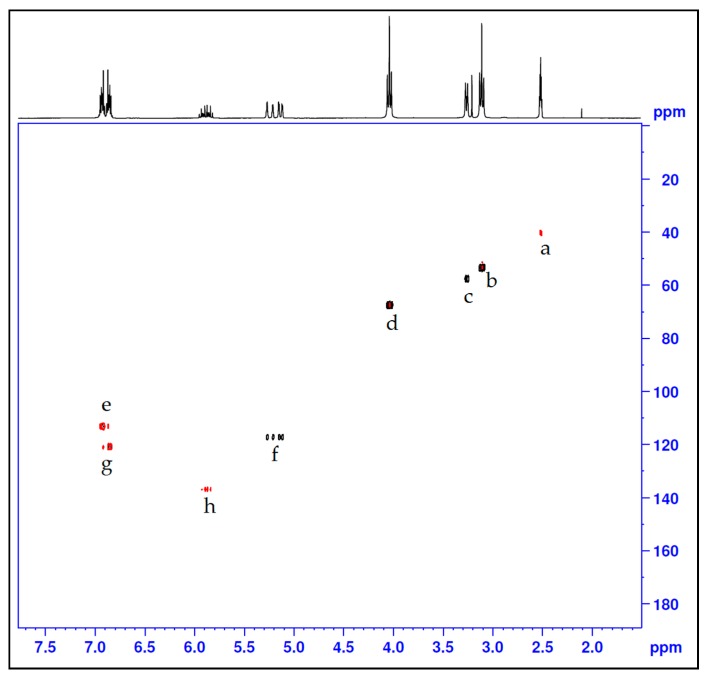
HSQC-NMR for the azacrown polyether macrocycle.

After chlorination of compound **2**, compound **3** was produced and had it displayed proton NMR spectra (Figure 2b) with the following shifts: δ ppm 2.88–2.99 (m, 4H), 3.30 (dt, *J* = 6.35, 1.36 Hz, 2H), 3.56–3.66 (m, 4H), 5.10–5.20 (m, 1H), 5.22–5.32 (m, 1H), 5.80–5.97 (m, 1H). The corresponding ^13^C-NMR data was as follows (Figure 3b): δ (ppm): 135.94 (C9) 116.88 (C10), 57.42 (C8), 55.89 (C2 & C5), 42.00 (C3 & C6) whereby we observed the significantly shifted signal of the carbon atoms attached to the chloride. On the other hand, ^1^H- and ^13^C-NMR signals (Figure 2c and Figure 3c) together with the corresponding long-range ^1^H-^13^C correlations (Figure 4) of the synthesized azacrown polyether confirmed the proposed structure. The signal for HDO (deuterated water) due to exchange of protons with the solvent deuteriums occurs at δ = 2.80 ppm as a singlet. Comparison of the ^1^H-NMR spectra of the precursor compounds and those of the azacrown polyether reveals that the new signals in the region between 6.81 ppm and 7.00 ppm are characteristic of the eight benzene protons (19, 20, 21, 22 and 23, 24, 25, 26). The rest of the proton signals can be assigned to the allyl fragments and the polyether ring cavity as already discussed in the precursor compounds. Integration of the signals for all the compounds (Table 2) confirms the assignments and proves the purity of the synthesized compounds. In ^13^C-NMR spectrum, the 8 chemical shifts for the crown polyether reveal 8 carbon environments (Figure 3c). Using the HSQC (^1^H-^13^C correlation) spectrum in Figure 4, the seven protonated carbon environments were assigned according to Table 3 while the unprotonated carbons environment (C8, C9, C17 and C18) was then assigned to δ = 148.88 ppm.

**Table 2:** Proton NMR signal integration for compounds **2**–**4**.

Compound	Proton Position	Integral	Multiplicity
**2**	9a	0.07	1
	10a & 10b	0.14	2
	2a, 2b, 6a, & 6b	0.28	4
	8a & 8b	0.14	2
	3a, 3b, 5a & 5b	0.28	4
**3**	9a	0.07	1
	10a & 10b	0.14	2
	3a, 3b, 6a & 6b	0.28	4
	2a, 2b, 5a & 5b	0.28	4
**4**	19, 20, 21, 22, 23, 24, 25 & 26	0.08	8
	28, 31	0.02	2
	29a, 32a	0.02	2
	29b, 32b	0.02	2
	2, 6, 11, & 15	0.08	8
	27 & 30	0.04	4
	3, 5, 12 & 14	0.08	8

**Table 3:** ^13^C-NMR signal assignments for the azacrown polyether.

Entry	Carbon Position	Chemical Shift (ppm)
a	Solvent (Acetone)	40.00
b	C3, C5, C12, C14	53.43
c	C27, C30	57.63
d	C2, C6, C11, C15	67.63
e	C20, C21, C24, C25	113.41
f	C29, C32	117.39
g	C19, C22, C23, C26	121.14
h	C28, C31	137.08
i	C8, C9, C17 and C18	148.88

**Figure 5:**
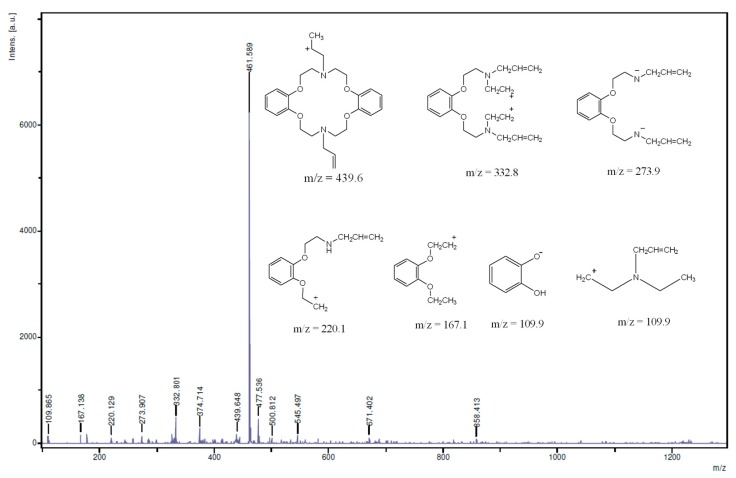
MS spectrum of the azacrown polyether macrocycle (solvent used; THF); (Inset are the important MS fragments of the macrocycle).

The NMR spectroscopy clearly revealed that the synthesis of the targeted molecule was achieved. There was no evidence revealing any impurities in the NMR spectra and the proton signal integration matched proton populations thus proving the purity of the molecule. Further evidences were however sought by mass spectroscopy (Figure 5) and elemental analysis (results for the latter are presented in the Experimental Section). The mass spectrum of the macrocycle revealed several peaks: some of them had *m*/*z* greater than the molecular mass of the synthesized compound suggesting that the ions may have resulted from the molecular recombination within the fragmentation process.

These peaks include *m*/*z* = 461.6, 477.5, 500.8, 545.5, 671.4, and 858.4. For the analyzed peaks we have proposed structures shown in Figure 5; they include *m*/*z* = 109.8, 167.1, 220.1, 273.9, 332.8, 374.7, 439.6. The detection of the molecular ion peak [M + H]^+^ for the azacrown polyether at *m*/*z* 439.6 confirmed its molecular mass which is 438.6 g/mol.

Also the detection of *m*/*z* = 109.8 suggests that the compound could possibly fragment into its starting reactant fragments—catechol and compound **3**—both of which have similar *m*/*z* values. The catechol ion fragment has been detected in a different crown ethers; dibenzo-18-crown-6 polyether [18]. Other important fragments observed at *m*/*z* 374.7, 332.8, 273.9, 220.1 and 167.1, correspond to the proposed structures suggesting the existence of compound **4**. The fragmentation pattern shows that the ether chains are fragile under the experimental conditions leading to most of the fragmentation patterns suggested. Similar hypothesis suggesting fragmentation patterns proceeding by the loss of ether chain segments are in accordance with other studies relating to the synthesis of benzo-15-crown-5 moiety [24]. Furthermore, allyl fragments were not detected in the MS spectrum.

### 2.2. Thermal Analysis of the Azacrown Polyether Macrocycle

**Figure 6:**
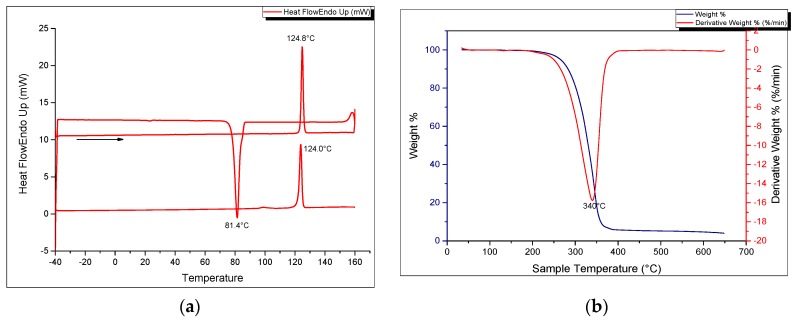
(**a**) DSC heating and cooling thermogram (**b**) TGA and DTG curves of the azacrown polyether macrocycle.

Figure 1a shows the SEM image of the solid-state crystalline morphology of the macrocycle. They are white, fibrous needle-like crystals. In terms of its thermal properties, the melting point and crystallization temperatures were determined by means of a heating and cooling scan in differential scanning calorimetry (Figure 6a). The crystallization temperature was 81.45 °C (±0.03) while the melting point was precisely determined to be 124.00 °C (±0.17) in the second heating run and could be verified using a melting point apparatus. The enthalpy of fusion (H_fus_) was calculated to be 40.63 kJ/mol while the crystallization enthalpy change was −36.27 kJ/mol. The sharp melting and crystallization temperatures are consistent with high purity of the synthesized compound. The thermal stability of the azacrown ether was also determined using TGA. The thermograms are shown in Figure 6b. The TGA data shows that there is only one major degradation step at 340 °C and it is accompanied by a complete weight loss of the whole molecule above 400 °C. The onset of degradation at 290 °C demonstrates that no decomposition occurs before its melting temperature at 124.0 °C.

## 3. Experimental Section

### 3.1. General Information

The melting point of the crown ether was initially determined using a B-540 Melting Point apparatus (Büchi, Flawil, Switzerland) equipped with capillary tubes and programmable temperature control. Differential scanning calorimetry was used as a main tool to confirm this m.p as well as the crystallization temperature using a DSC-8500 instrument (Perkin-Elmer, Rodgau, Germany) with samples (4–5 mg) sealed in high-pressure steel pans. The DSC temperature ramp was 10 °C/min under N_2_ flow of 20 mL/min while TGA data was recorded on a Pyris 1 thermo-gravimetric analyzer (Perkin-Elmer) at 10 °C/min under N_2_ flow. ^1^H- and ^13^C-NMR as well as 2D-NMR were recorded using an Avance DPX 300 MHz nuclear magnetic resonance spectrometer (Bruker, Wien, Austria) with acetone-*d*_6_ as solvent. For elemental analysis, dried samples were analyzed on a Vario elemental analyzer (Elementar Analysensysteme GmbH, Hanau, Germany). FT-IR was performed on ATR crystal mode on a FT-IR spectrometer 65 (Perkin Elmer, Waltham, MA, USA) using 32 scans at a resolution of 2 cm^−1^. The electronic spectra of the macrocycle polyether were recorded in the region 700–200 nm using a PerkinElmer double beam model Lambda 35 spectrometer with a quartz cell of 1 cm path length in cyclohexane. Bis(2-hydroxyethyl)amine, allyl bromide, thionyl chloride and catechol were purchased from Sigma-Aldrich (Munich, Germany) and used without further purification. Potassium carbonate, sodium hydroxide pellets, *n*-butanol, acetone, methanol, diethyl ether and all other solvents were reagent grade and used without further purification.

### 3.2. Synthesis of the Azacrown Polyether Macrocycle

#### 3.2.1. Synthesis of 2,2′-(Prop-2-en-1-ylimino)diethanol (**2**)

Bis(2-hydroxyethyl)amine (105.14 g, 1.0 mol) was mixed with potassium carbonate (1.00 mol) in a three-neck round-bottomed flask under mechanical stirring. 3-Bromopropene (allyl bromide, 121.0 g, 1.00 mol) was added dropwise by means of a dropping funnel over a period of 1–2 h. The reaction between the bis(2-hydroxyethyl)amine and the allyl bromide was allowed to proceed for 6 h at 170 °C under reflux. The reaction mixture was cooled to 70 °C, dissolved in ethanol (100 mL) and filtered. The filtered solution was distilled under vacuum and collected at 111 °C/1000 mbar. Yield 110.8 g (76.3%), as a yellowish viscous oil. ^1^H-NMR (acetone-*d*_6_) δ (ppm): 2.65 (t, *J* = 5.74 Hz, 4H), 3.21 (dt, *J* = 6.42, 1.32 Hz, 2H), 3.57 (t, *J* = 5.74 Hz, 4H), 5.08–5.15 (m, *J* = 19.9 Hz, 1H), 5.20 (dq, *J* = 17.22, 1.71 Hz, 1H), 5.90 (m, *J* = 17.00, 10.35, 6.57, 6.57 Hz, 1H). ^13^C-NMR (acetone-*d*_6_) δ (ppm): 135.27 (C9), 116.42 (C10), 59.50 (C2, 6), 57.78(C8), 56.08 (C3, C5); IR (ν/cm^−1^): 3080 (alkene =C-H stretch), 2947 and 2828 (methylene stretch), 1642 (C=C stretch), 1450, (aliphatic C-H bend), 1354 and 1141 (C-N stretch aliphatic amines), 1060 (C-OH stretch), 921 (=C-H bend alkenes); Anal. Cacl. for C_7_H_15_NO_2_ (145.2 g/mol): C, 57.90; H, 10.41; N, 9.65; O, 22.04. Found. C, 57.60; H, 10.64; N, 9.57.

#### 3.2.2. Synthesis of *N,N*′-Bis(2-chloroethyl)prop-2-en-1-amine (**3**)

The chlorinated product *N,N*′-bis(2-chloroethyl)prop-2-en-1-amine (**3**) was obtained by reaction of **2** with thionyl chloride in chloroform solution. In details, a 1.03% *w*/*v* solution of **2** (102.80 g equivalent to 0.71 mol) in chloroform (100 mL) was carefully added by means of a dropping funnel to ice-cooled (warning!: the reaction is very exothermic) thionyl chloride (1.42 mol, 168.76 g) contained in a two-neck round-bottomed flask. After all the funnel contents had been added, the ice bath was replaced with a water bath set at 40 °C and the reaction was continued at reflux for 4 h with magnetic stirring. Then, the reactor flask was cooled to room temperature (~23 °C) and distilled to remove excess SOCl_2_ and chloroform. The entire contents of the reaction were then put under an ice bath and cooled to 0 °C. NaOH (2.5 M, 100 mL) was then added to the reactor. The pH of the mixture was adjusted to neutral with saturated solution of sodium carbonate, leading to two immiscible liquid phases. The oily layer was separated from the aqueous one by mean of a separatory funnel and afterwards distilled under pressure. A clear liquid distillate was obtained at 78–79 °C/1000 mbar with a total yield of 91.6% (118.15 g). The *N,N'*-bis(2-chloroethyl)prop-2-en-1-amine product was designated as compound **3**. It was used immediately for the next reaction or stored in a brown glass bottle and kept under 4 °C. ^1^H-NMR (acetone-*d*_6_) δ ppm 2.88–2.99 (m, 4 H), 3.30 (dt, *J* = 6.35, 1.36 Hz, 2 H), 3.56–3.66 (m, 4 H), 5.10–5.20 (m, 1 H), 5.22–5.32 (m, 1 H), 5.80–5.97 (m, 1 H). ^13^C-NMR (DMSO-*d*_6_) δ ppm: 135.94 (C9), 116.88 (C10), 57.42 (C8), 55.89 (C3, C6), 42.03 (C2, C5); IR (ν/cm^−1^): 3080 (alkene =C-H stretch), 2957 and 2816 (methylene stretch), 1642 (C=C stretch), 1450, (aliphatic C-H bend), 1257 (C-H_wag_ of the -CH_2_Cl), 1354 and 1107 (aliphatic amine C-N stretch), 921 (alkene =C-H bend), 740 (C-Cl stretch); Anal. Calc. for C_7_H_13_Cl_2_N (182.1 g/mol): C, 46.17; H, 7.20; N, 7.69; Cl, 38.94. Found. C, 46.13; H, 7.48; N, 7.65.

#### 3.2.3. Synthesis of *N,N*′-Diallyl-7,16-diaza-1,4,10,13-tetraoxa-dibenzo-18-crown-6 (**4**)

A dry, 2-L, three-necked flask was fitted with a reflux condenser, a 250-mL, pressure-equalizing dropping funnel, and a nitrogen inlet tube. The flask was charged with catechol (110.11 g, 1.00 mol) and *n*-butanol (300 mL) before stirring was started, and sodium hydroxide pellets (40 g, 1.00 mol) were added. The mixture was rapidly heated to reflux at ~115 °C thereby producing the corresponding salt of the catechol (sodium catecholate). Afterwards, a solution of *N,N*-bis(2-chloro- ethyl)prop-2-en-1-amine (3, 91.05 g, 0.50 moles) in *n*-butanol (90 mL) was added dropwise with continuous stirring and heating over a 2 h period. After the resulting mixture had been refluxed with stirring for an additional hour, it was cooled to 90 °C and another 40 g of NaOH pellets were added. The mixture was further refluxed for 30 min, and another solution of **3** (91.05 g 0.50 moles) in *n*-butanol (90 mL) was added dropwise with continuous stirring and heating over 2 h period. Finally, the reaction mixture was refluxed for 21 h, then acidified by the dropwise addition of concentrated hydrochloric acid (7 mL) The reflux condenser was replaced with a distillation column and approximately 250 mL of solvent was distilled from the mixture. As the distillation was continued water was added to the flask from the dropping funnel at a sufficient rate to maintain a constant volume in the reaction flask. The distillation was continued until the temperature of the distilling vapor reached ~100°, and the resulting slurry was cooled to room temperature to precipitate. The solid was dissolved in acetone (250 mL) and stirred to coagulate it and then filtered off on a glass frit. The residual crude was stirred with 1 L of water, decanted and recrystallized three times from acetone and dried at 60 °C to yield 91.38 g (42.0%) of white fibrous crystals, m.p. 124 °C; UV (cyclohexane) λ_max_ (log ε) 236 (2.82), 278 (2.55) nm; ^1^H-NMR (acetone-*d*_6_) δ ppm 3.22 (t, *J* = 6.07 Hz, 8 H), 3.34 (dt, *J* = 6.22, 1.44 Hz, 4 H), 4.12 (t, *J* = 6.23 Hz, 8 H), 5.09–5.20 (m, 2 H), 5.20–5.34 (m, 2 H), 5.85–6.04 (m, 2 H), 6.81–7.00 (m, 8 H); ^13^C-NMR (acetone-*d*_6_) δ ppm: 148.88 (C28, C31), 137.08 (C29, C32), 121.14 (C8, C9, C17, C18), 117.39 (C19, C22, C23, C26) 113.41 (C20, C21, C24, C25) 67.63 (C2, C6, C11, C15), 57.63 (C27, C30), 53.4 (C3, C5, C12, C14); IR (ν/cm^−1^): 3068 (aromatic C-H stretch), 2926 and 2855 (methylene stretch), 1634 (C=C stretch), 1596, 1496 and 1456 (C=C aromatic ring stretch), 1334 and 1120 (C-N stretch), 1238 (aryl-O-CH2 stretch), 1024 (in-plane C-H bend), 728 (out-of-plane C-H bend); Anal. Calc. for C_26_H_34_N_2_O_4_ (438.6 g/mol): C, 71.21; H, 7.81; N, 6.39; O, 14.59. Found C, 71.03; H, 7.82; N, 6.22.

## 4. Conclusions

In this paper we have described an optimized and scaled-up synthesis of the bibracchial lariat azacrown ether *N,N*′-diallyl-7,16-diaza-1,4,10,13-tetraoxa-dibenzo-18-crown-6. The production scale-up to one-mole batch of reactants is demonstrated in yields up to 35%–45%. This is a large improvement compared to the prior art, where batch sizes of ~0.1 moles and yields of about 3%–6% are obtained. The improvement is made possible by implementing a two-step addition of strong base under anhydrous conditions to improve the cyclization process. The combination of neutralization of the reaction mixture and distillation of the excess solvent and its subsequent replacement with distilled water was key in the formation of a high purity precipitate that crystallizes from acetone. The convergent results obtained by elemental analysis, FTIR, UV-Vis, NMR MS, and thermal analysis allowed us to confirm the structural formula of the crown ether and its purity. With the availability of a new synthesis on a larger scale and the good yields of this azacrown ether, its use in supramolecular chemistry and ion-selective complexation is made possible.

## Supplementary Materials

Supplementary materials can be accessed at: http://www.mdpi.com/1420-3049/21/2/171/s1.
