# Supplementary material for: Synthesis and Characterization of Macrocyclic Polyether N,N′-Diallyl-7,16-diaza-1,4,10,13-tetraoxa-dibenzo-18-crown-6
Source: Molecules. 2016 Jan 29;21(2):171. doi: 10.3390/molecules21020171 (PMC6274274; doi:10.3390/molecules21020171)
Supplement: Supplementary file 1 [file molecules-21-00171-s001.pdf]

Supplementary Materials: Synthesis and Characterization of Macrocyclic Polyether *N,N′*-diallyl-7,16-diaza-1,4,10,13-tetraoxa-dibenzo-18-crown-6

Julius Toeri and Marie-Pierre Laborie

Figure S1a shows the characteristic secondary N-H amine band maximum at 3298 cm^{-1} together with its aliphatic secondary C-N amine band at 1127 cm^{-1} for the starting bis(2-hydroxyethyl)amine. This secondary N-H amine band disappears in **2**, **3** and **4** and new C-N tertiary amine band formed exhibit at 1141 cm^{-1} for compound **2**; 1107 cm^{-1} for **3** while in **4**, the same band appear at 1120 cm^{-1} all being indicative of the occurrence of the allyl reaction [1,2]. The existence of the appended allyl fragment in compound **2**, **3** and **4** is also confirmed by the presence of the bands at 3080 and 3006 (b,c) and 3068 and 3006 cm^{-1} (d) which are typical of =C-H stretches, and the moderate band at 1642 cm^{-1} due to stretching vibration of the C=C double bond. The infrared spectrum of the azacrown polyether showed new bands at 1596, 1504 and 1456 cm^{-1} attributable to C=C aromatic stretch of the catechol fragments. Similar data representing benzene ring signals were also reported by Correa and Scott [3].

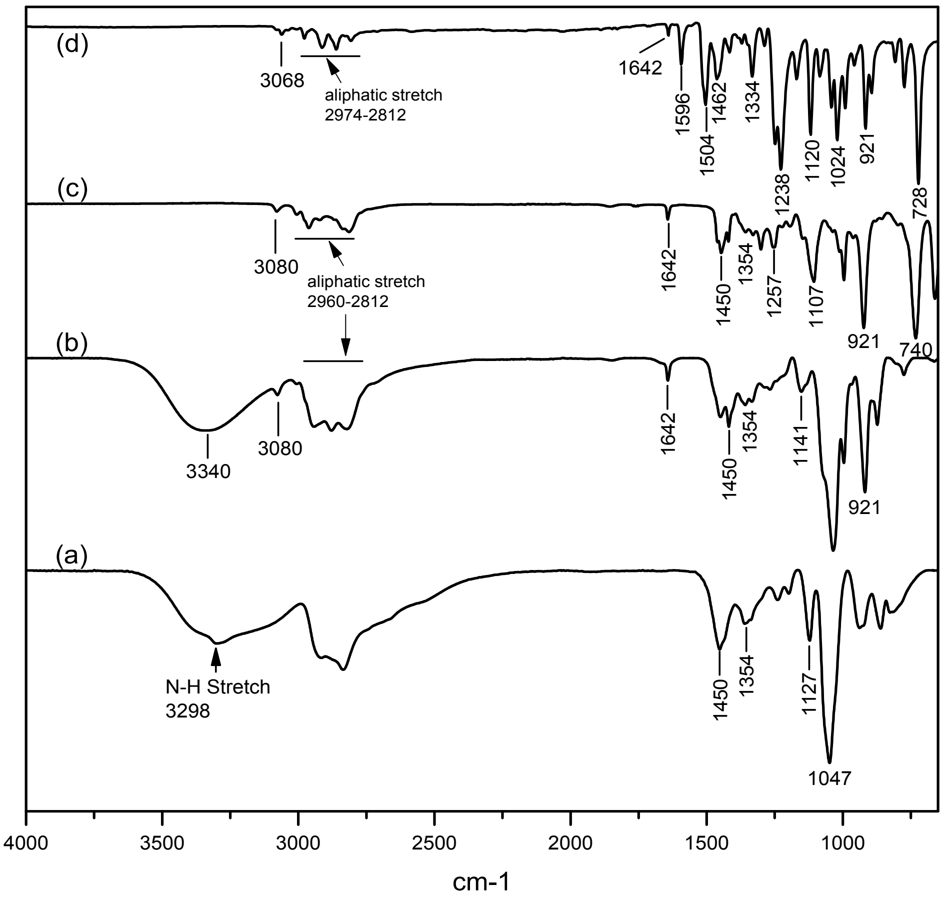

Figure S1. ATR-FTIR spectra of: (**a**) the starting bis(2-hydroxyethyl)amine **1**; (**b**) 2,2′-(prop-2-en-1-ylimino)diethanol **2** obtained after allylation; (**c**) *N,N*-bis(2-chloroethyl)prop-2-en-1-amine **3** obtained after chlorination; and (**d**) *N,N′*-diallyl-7,16-diaza-1,4,10,13-tetraoxa-dibenzo-18-crown-6 **4**.

The UV-Vis spectrum of **4** (Figure 3) was recorded in cyclohexane (2.28 × 10^{-4} M). The product has two UV maxima of 236 nm (ε 12,368) and 278 nm (ε 11,184) both of which are $\pi \rightarrow \pi^*$ and can be attributed to the benzene fragment of the crown ether cavity. A related macrocycle, dibenzo-18-

crown-6 polyether, which was synthesized by Pedersen [4], and has all-oxygen crown ether cavity was reported with similar absorption values hence supporting the possibility that the two signals arise as a result of the presence of benzene ring.

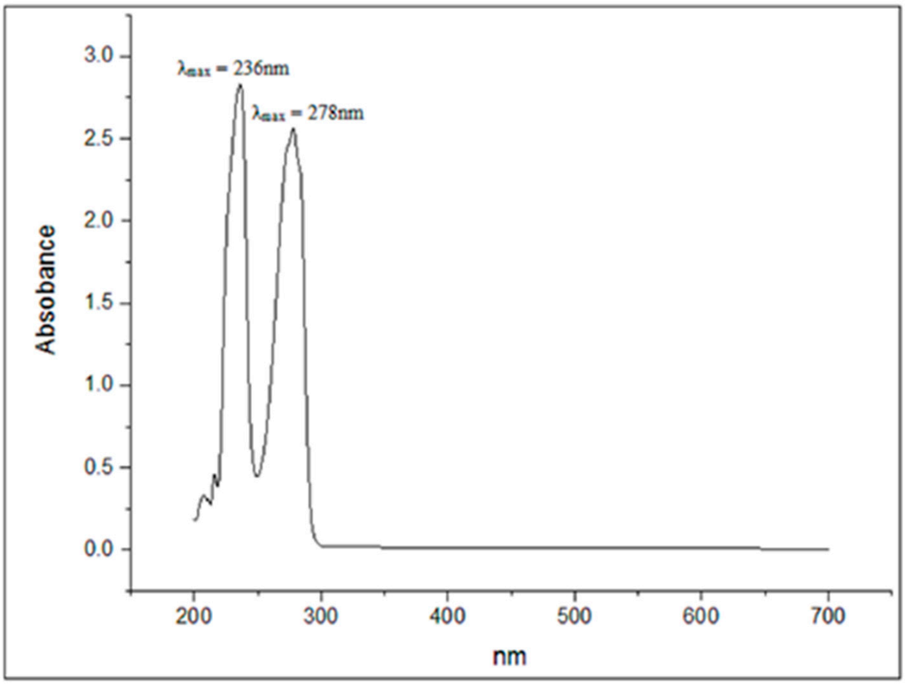

Figure S2. UV-Vis spectrum of the azacrown polyether in cyclohexane.

References

1. Pretsch, E.; Bühlmann, P.; Affolter, C. *Structure Determination of Organic Compounds: Tables of Spectral Data*, 3rd ed.; Springer: Berlin, Germany; 2000.
2. Silverstein, R.M.; Webster, F.X. *Spectrometric Identification of Organic Compounds*, 6th ed.; Wiley: New York, NY, USA, 1998.
3. Correa, W.H.; Scott, J.L. Synthesis and Characterisation of Macrocyclic Diamino Chiral Crown Ethers. *Molecules* **2004**, *9*, 513–519.
4. Pedersen, C.J. Macrocyclic polyethers: Dibenzo-18-crown-6 polyether and dicyclo-18-crown-6 polyether. *Org. Synth.* **1972**, *52*, 66, doi:10.15227/orgsyn.052.0066.
